# The murine lung microbiome in relation to the intestinal and vaginal bacterial communities

**DOI:** 10.1186/1471-2180-13-303

**Published:** 2013-12-28

**Authors:** Kenneth Klingenberg Barfod, Michael Roggenbuck, Lars Hestbjerg Hansen, Susanne Schjørring, Søren Thor Larsen, Søren Johannes Sørensen, Karen Angeliki Krogfelt

**Affiliations:** 1 Statens Serum Institut, Artillerivej 5, 2300 Copenhagen S, Denmark; 2 National Research Centre for the Working Environment, Lersø Parkallé 105, 2100 Copenhagen O, Denmark; 3 Department of Biology, Microbiology, University of Copenhagen, Universitetsparken 15, 2100 Copenhagen O, Denmark

## Abstract

**Background:**

This work provides the first description of the bacterial population of the lung microbiota in mice. The aim of this study was to examine the lung microbiome in mice, the most used animal model for inflammatory lung diseases such as COPD, cystic fibrosis and asthma.

Bacterial communities from broncho-alveolar lavage fluids and lung tissue were compared to samples taken from fecal matter (caecum) and vaginal lavage fluid from female BALB/cJ mice.

**Results:**

Using a customized 16S rRNA sequencing protocol amplifying the V3-V4 region our study shows that the mice have a lung microbiome that cluster separately from mouse intestinal microbiome (caecum). The mouse lung microbiome is dominated by *Proteobacteria, Firmicutes, Actinobacteria, Bacteroidetes* and *Cyanobacteria* overlapping the vaginal microbiome. We also show that removal of host tissue or cells from lung fluid during the DNA extraction step has an impact on the resulting bacterial community profile. Sample preparation needs to be considered when choosing an extraction method and interpreting data.

**Conclusions:**

We have consistently amplified bacterial DNA from mouse lungs that is distinct from the intestinal microbiome in these mice. The gut microbiome has been extensively studied for its links to development of disease. Here we suggest that also the lung microbiome could be important in relation to inflammatory lung diseases. Further research is needed to understand the contribution of the lung microbiome and the gut-lung axis to the development of lung diseases such as COPD and asthma.

## Background

Studies of the lung microbiome by culture independent techniques and its impact on lung immunity is a relatively new field and may contribute to new advances in understanding respiratory diseases [1]. Healthy human lungs have up until recently been considered to be sterile by culture-based techniques, but now new evidence have identified microbial communities both in healthy humans and in those with disease [2-4]. The human microbiome project [5] did not originally include the lungs, but recently the Lung HIV Microbiome Project has published the first results in this field [6,7]. Investigations into lung microbiology and lung immunity in humans is limited largely because of technical, ethical considerations and small samples sizes, whereas the use of animal models can provide novel information useful in investigations into the importance of lung microbiome in the development of lung immunology. Effective utilization and development of animal models have recently been identified as one of the most important challenges in future lung microbiome research by the NIH [8]. Whereas many studies have focused on the gut microbiome and its impact on among others lung immunity and asthma, little work has been performed to examine the contribution of the lung microbiome on the pathogenesis of pulmonary diseases. Especially in inflammatory lung diseases such as asthma and COPD, the local microbiome may play an important role in the pathogenesis. The technical challenges related to the novel culture-dependent techniques include consistent extraction of useful DNA, the development of PCR methods and sampling methods for the less abundant bacterial load of the lungs.

We hypothesized that the problems with getting bacterial DNA from lungs was due to the presence of host DNA in the extractions. In this study, we have investigated the bacterial community from lungs of 20 mice using rDNA amplicon 454 pyrosequencing. We also performed a conventional cultivation study of 10 mouse bronchoalveolar lavage (BAL) fluids on different agar plates. Sampling methods and DNA extraction protocols were investigated systematically: one BAL sample still containing mouse cells (BAL-plus) and one BAL sample, where the mouse cells were removed (BAL-minus) by cytospin. The bacterial communities in BAL samples were compared using DNA extractions from washed lung tissue, caecum samples and vaginal flushing. We chose to include vaginal samples for two major reasons. The vaginal microbiome of BALB/c has not previously been described and could have influence on microbial “priming” and transfer from mother to pup. In this study, it also serves a reference sample from a different mucoid epithelium than lung. The bacteria were classified by their sequence into Operational Taxonomic Units (OTU). An OTU is an approximation to taxonomy derived from classical cultivation techniques.

We demonstrate the use of this methodology and describe an uncultivable lung and vaginal microbiome in mice that are diverse and distinct from caecal microbiome. Our results provide a basis for further studies into the lung microbiome in culture negative BAL fluids in mouse models of inflammatory lung diseases suggested by descriptive human studies.

## Methods

### Mice and sample collection

BALB/cJ female mice, reared together (Taconic M&B, Ry, Denmark), 7 weeks old, body weight 18–22 g, were randomly distributed and housed 10 animals per cage (425 × 266 × 150 mm) with tap water and food (Altromin no 1324 Brogaard Denmark) provided ad libitum. Light/dark cycles were at 12 hours and room temperature and relative humidity was kept at 19-22°C and 40-60%, respectively. Animals were handled by the same two animal technicians and conditioned in our animal facility for two weeks before use.

The BAL procedure was performed as previously described with minor modifications [9]. We inserted sterile tube (Insyte, BD, Denmark) for each mouse and lungs were flushed two times with 0.8 mL pyrogenfree saline (0.9%)(Fresenius Kabi, Denmark) and the recovered fluids were pooled (LF-plus). For the BAL samples without mouse cells (BAL-minus) the BAL fluid was spun at 400 g for 10 min a 4°C collecting the supernatant. All the BAL samples were frozen at -80°C.

Lung tissue was collected using one, chlorine [10] and heat treated sterile scissors, per animal cutting the distal tip of the left lung after the BAL procedure. Tissues were snap-frozen in liquid nitrogen.

Vaginal fluid samples were performed by inserting a sterile pipette tip into the vaginal space flushing 3 times back and forth with 30 μL pyrogenfree infusion saline (0.9%) (Fresenius Kabi, Denmark) and frozen at -80°C.

As the last procedure, the caecum samples were taken from the animals. With a sterile scissor the caecum was cut open and approximately 50 mg of caecal matter was removed using sterile plastic loops directly into cryo tubes and snap frozen in liquid nitrogen. All protocols were approved by the Danish Animal Experiments Inspectorate.

### Bacterial identification by culturing

Mouse BAL fluids, 200 μL per mouse, were cultivated on general growth media blood agar 5% (SSI, Denmark) and Chocolate Agar (SSI, Denmark) for fastidious bacteria and incubated at 37°C for 24 hours. Another set of plates with selective media was incubated under micro aerophilic conditions (5%CO_2_, 3%H_2_, 5%O_2_ and 87%N_2_) at 37°C for 48 hours [11]. The bacterial colonies were subjected to routine identification by the Vitek2 system (Bio Mérieux, France).

### DNA extraction and PCR

Isolation of bacterial DNA from frozen BAL or vaginal samples was done using Qiagen spin protocol (Qiagen, DNA mini kit Denmark) for body fluids with the following modifications: Tubes were thawed and centrifuged at 16.000 g for 5 min to spin down all the bacteria. The supernatant was discarded and the bacterial pellet was resuspended with 450 μL lysis buffer. Forty-five μL proteinase K and add 0.3 mL 0.1 mm zirconium/silica beads (Techum, Sweden) were added. Proceed with bead beating step using TissueLyser (Qiagen, Denmark) for 6 min at 30 Hz. [12]. Lysis was performed by incubating in heat block at 56°C for 10 min. and then at 95°C for 7 min. Proceed with protocol for body fluids from step 5. At the elution step, the AE buffer is preheated to 65°C and DNA elution is performed with 100 ul with 3 minutes incubation at room temp before final spin. Isolation of bacterial DNA from frozen caecal or tissue was done using Qiagen spin protocol for detection of pathogens from stool (Qiagen, DNA mini stool kit Denmark) with the following modifications: Add 1.4 ml of the ASL buffer and perform bead beating, lysing and eluding as describe above for body fluids. For tissues samples, chlorine [10] and heat sterilized 3 mm steel bead (Qiagen, Denmark) was added to the samples along with the zirconium/silica beads for extra tissue disruption.

### 16S sequencing

Amplicon libraries of the 16S rRNA gene of caecum, BAL and vaginal samples were prepared with two PCR reactions. In the first PCR, a 466 bp long fragment covering the variable region V3 and V4 of the 16S rRNA gene, was amplified with AccuPrime™ Pfx DNA Polymerase and the bacteria and archaea specific primers 341 F and 806R (Table 1). The reaction started with an initialization at 94°C for 2 min, followed by 44 cycles of denaturation at 94°C for 20 sec, annealing at 56°C for 30 sec. and elongation at 68°C for 40 sec. The reaction was completed with a final elongation at 68°C for 5 min. Due to the low DNA (<0.5 ng × μL^-1^) concentration in the samples we needed to increase the cycle number above the standard of 30–35. This adjustment highly increased the risk of amplifying contamination from extraction buffer and other experimental used liquids. To minimize this possibility we chose the lowest cycle number with a clear amplification band in the agarose gel and no signals of negative controls from BAL procedure for DNA purification.

**Table 1:** Primers

**Primer**	**Sequence**	**Reference**
27 F	5′AGAGTTTGATCMTGGCTCAG-3′	[13]
341	5′-CCTAYGGGRBGCASCAG-3′	[14,15]
806	5′-GGACTACNNGGGTATCTAAT-3′	[14,15]
TitA_341F	5′-CGTATCGCCTCCCTCGCGCCATCAG-TAG-CCTAYGGGRBGCASCAG-3′	[16]
TitB_806R	5′-CTATGCGCCTTGCCAGCCCGCTCAG-GGACTACNNGGGTATCTAAT-3′	[16]
1492R	5′-GGTTACCTTGTTACGACTT-3′	[13]

In the second PCR the adaptors were attached to the amplicon library elongating the fragment towards 526 bp with the primer TitA_341F and TitB_806R. The same reaction conditions of PCR I were applied in PCR II with a reduced cycle number of 15.

Initially we tried to apply the same procedure for the lung tissue samples but unspecific bands after gel-electrophoresis made it impossible to select the correct fragment size. To overcome this problem we chose the primer 27 F and 1492R amplifying the entire 16S rRNA gene which appeared to be more specific. The PCR I conditions were the same as mentioned above except that the annealing temperature was reduced to 55°C and the cycle number to 40. In this perspective the Tag-PCR reaction with TitA_341F and TitB_806R provided the selection for V3 and V4 as well as attaching the adaptors to the amplicons.

### Statistical analysis and bioinformatics

The 16S rRNA gene sequences obtained from one half a plate of a 454 - Roche - Titanium pyrosequencing run were quality filtered, trimmed and split into the corresponding animal samples with the Qiime pipeline version 1.6.0 using the default settings [17]. We considered only sequences with a minimum length of 250 bp. Chimeras were removed by UCHIME [18]. The operational taxonomic units (OTU) were picked *de novo* and clustered at 97% sequence similarity. The taxonomy was assigned using RDP classifier (bootstrap threshold 0.8) greengenes as reference database [19].

For statistical analysis, raw data were transferred into the open source statistical program “R” [20]. The non-parametric Wilcoxon test (W) evaluated variations of alpha diversity between two variables. We used the non-parametric Kruskal-Wallis-test when comparing more than 2 variables (KW). Dissimilarities in OTUs abundance between the samples were explained by KW and the sample clustering of the OTU count based Bray-Curtis distance metric were examined by the analysis of similarity (anosim).

## Results

To determine the airway bacterial microbiota of the BALB/cJ mouse model based on 16S rDNA gene sequencing, we have compared sequences found in the lungs with three different approaches, to sequences found in corresponding vaginal and caecal samples.

### Over all sequence quality and results from all sample types

We generated a total of 908256 sequences. After quality filtering and chimera check, 27% of sequences were removed and 660319 sequences were further processed for OTU picking (sequences ranged between 3530 up to 31638 per animal sample). The *de novo* OTU clustering revealed 6487 OTUs. The OTU table was randomly subsampled to avoid differences based on sequencing effort leaving 3318 OTUs for further analysis (Rarefaction curve are shown in Additional file 1: Figure S5).

**Figure 1:**
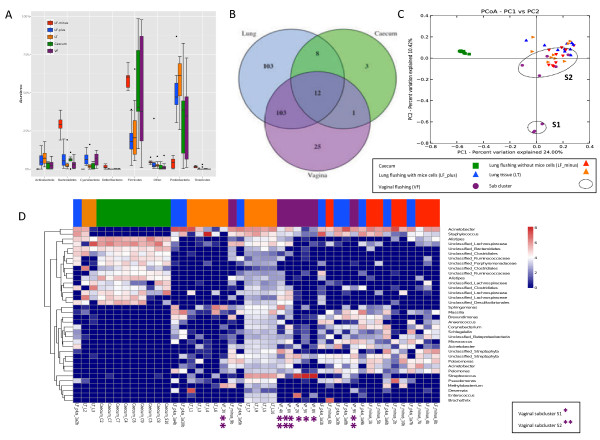
**Community composition. (A)** Distribution of Phyla between sample types. LF-plus bronchoalveolar lavage (BAL) fluids and LF-minus is BAL where the mouse cells have been removed. LT is lung tissue and VF is vaginal flushing, **(B)** Venn diagram of identified shared and unique genera from each sampling site. All the lung type samples are considered here as one. (complete list shown in Additional file 3: Table S4), **(C)** The PcoA plot is generated of the Bray-Curtis dissimilarity metric based on OTU counts and explains the largest variance between all samples (PCoA plot 1vs 3 and PCoA plot 2 vs. 3 are attached in Additional file 4: Figure S4), **(D)** Heat map of even subsampled OTU table. The dendrogram is two sited hierarchal clustered by abundance dissimilarity and the data are log transformed. Shown are only taxa, which counted for at least 0.5% of the generated sequences. The x-axis clusters the animal samples and the y-axis the taxonomical information. * marks Vaginal subcluster S1 and ** subcluster S2.

We found a total of 19 bacterial phyla in the samples analysed. The most dominant (>0.5% abundance) phyla observed were *Acidobacteria, Actinobacteria, Bacteroidetes, Firmicutes, Proteobacteria* and *TM7.* The difference in bacterial composition at the phylum level between sampling sites is shown in Figure 1A.

In Additional file 2: Table S2 we have listed all the bacteria that were found, which were unique for the lung samples and which were shared between sampling sites.

### The bacterial sequences of the lung samples

If we only look at the lung samples, the most dominant lung phyla found were *Proteobacteria, Firmicutes, Actinobacteria, Bacteroidetes* and *Cyanobacteria.* Additionally we observed *Fusobacteria* and *Cyanobacteria* in the lung and vaginal samples.

In order to highlight phyla variations in the lung community compared to vaginal and caecal communities, we first we took the three lung sample types: bronchoalveolar lavage fluids (BAL-plus), and BAL-minus, where the mouse cells have been removed by a spin protocol and finally lung tissue from the distal tip of the lung and considered them as one ecological community. In this lung community profile, *Actinobacteria*, and *Proteobacteria* were clearly more abundant than in the caecum community (KW, p < 0.0001).

Then, looking at the differences between the three lung sample types, *Firmicutes* appeared (KW, p < 0.05) more abundant in lung tissue (57%) than in BAL samples (20%). The *SR1* bacteria were found only in BAL-minus and Lung tissue samples, but *Tenericutes* was observed in all samples, except in the vaginal samples. Other phyla observed below 0.5% abundance were *Chloroflexi, Deinococcus-Thermus, Fibrobacteres, Gemmatimonadetes, OD1, OP10, Planctomycetes, Verrucomicrobia,* and *WS3*.

Comparing lung sampling methods we also found a significant variation for *Actinobacteria* and *Cyanobacteria,* which were largely abundant in both type of BAL communities relative to the lung tissue samples (KW, p < 0.05). At phylum level, the composition of the lung tissue samples appeared to be very similar to the vaginal samples except for a larger abundance of *Cyanobacteria* in vaginal samples (KW, p < 0.05).

### Bacterial sequences of the caecum

Looking at the caecum samples, they contained more *Firmicutes* and *Bacteroidetes* KW, p < 0.0001) than the lung samples and *Acidobacteria* and *Cyanobacteria* were absent. The phylum *Bacteroidetes* (29%) appeared to be the second most abundant after the *Firmicutes* (59%). The vaginal and the caecal communities only had *Ruminococcus* in common, a genus that was not observed in the lung microbiota. Three genera were found in caecal samples alone; *Robinsoniella, Parasutterella* and *Ramlibacter*. The low numbers of genera detected in the caecal samples is due to the depth of taxonomic information obtained for these particular OTU sequences towards the consensus lineage of the database.

### Overlapping genera

For an overview comparison between the different sample types, we have merged the results found in the different lung communities and displayed the overlapping generawit hcaecum and vagina in a venn diagram. This diagram reflects 255 identified genera (summarized in Additional file 3: Table S4), that covers 76% of the sequences from BAL-plus, 68% from BAL-minus, 66% of vaginal and lung tissue community and 27% of sequences assigned to the caecum community (Figure 1B).

Lung samples, vaginal and caecum samples shared the 12 core genera *Bacteroides, Barnesiella, Odoribacter, Alistipes, Mucispirillum, Lactobacillus, Streptococcus, Peptoniphilus, Roseburia, Anaerotruncus, Oscillibacter, Pseudomonas*. We observed *Parabacteroides*, *Eubacterium*, *Marvinbryantia*, *Butyricicoccus*, *Papillibacter*, *Bosea, Anaeroplasma*, lung and caecum. The pulmonic and vaginal community shared 103 genera (Additional file 3: Table S4). Additionally *Akkermansia* was also found in the lung but only in one caecum sample in the raw data set.

### Variability in community composition between samples obtained from the same sampling site (Beta_diversity)

To make a sample to sample comparison and illustrate the variation between our mice we have performed a principle coordinate analysis (PCoA) based on the Bray-Curtis dissimilarity between OTU count metric PCoA plot (Figure 1C), which explains the largest variance between all samples (Additional PCoA 2 and 3 are found in Additional file 4: Figure S4).

The caecal samples cluster together at a significant distance from lung and vaginal communities, confirmed by the analysis of similarity, anosim (R = 0.673, p = 0.001)

The dissimilarity between the three lung communities was found to be little due to strong cluster overlap (anosim, R = 0.09, p = 0.05) when comparing only the lung distances.

We found large variation within the vaginal samples resulting in a division into subcluster 1 (S1), containing animal vaginal sample 8, 5 and 2, and subcluster 2 (S2), vaginal sample 3,4,6,9 and 10 (anosim, R = 0.72, p = 0.001). The separation is clearly shown in PCoA1 (Figure 1C) and PCoA3 (Additional file 4: Figure S4). Those samples that grouped into S1 were found to be less similar to caecum and lung communities, whereas samples grouping into S2 appeared more closely related to the lung microbiota.

A more detailed description of the taxa responsible for distinguishing bacterial communities in the lung, caecum and vagina is demonstrated using a heatmap dendrogram (Figure 1D).

We removed from the subsampled OTU table all observations accounting for less than 0.5% of the generated sequences to visualize the taxa with main impact on the community profile. This method provides maximal taxonomic resolution of each individual animal sample and directly reflects the PCoA plots since both analyses are based on OTU count dissimilarities.

For the caecum samples, 27% could be assigned to a taxonomic genus as mentioned before and the sequences belonged to *Alistipes* (16%) *Anaeroplasma* (1.5%) and a 22 genera listed in Additional file 3: Table S4. We observed a better taxonomic resolution on the family level, were 77% of the reads were successful assigned. The three major families in the caecum were *Lachnospiraceae* (33.8%), *Ruminococcaceae (15.3%)* and *Porphyromonadaceae* (7.9%)*.*

Vaginal samples within S1 contained between 56-97% of *Streptococcus,* while vaginal samples within S2 only had 0.2 – 10% of the gram-positive bacterium, explaining why here appears to be such a distinction between the S1 and S2 groups. In addition to *Streptococcus*, notable contributions from *Acinetobacter* (6.2%), *Sphinogmonas* (3.3%), *Enterococcus* (3.1%), and *Polaromonas* (1.8%) were also observed in the vaginal community.

All lung samples had representative sequences from genera including *Staphylococcus* (8.3%) *Massilia* (2.6%), *Corynebacterium* (2.2%), *Pseudomonas* (2.53%), *Streptococcus* (2.3%) and *Sphingomonas* (1.7%) without significant variation (KW, p > 0.05).

Even though the beta diversity measure indicated that there were minimal differences between the lung communities sampled using different methods, six major genera varied significantly (KW, p < 0.05). *Acinetobacter*, *Pelomonas*, and *Schlegella* were more abundant in the BAL-plus samples in comparison to the BAL-minus or the lung tissue samples. *Arcobacter*, and *Polaromonas* were highly associated with BAL-minus, whereas *Brochothrix* was only found in the lung tissue samples.

### Richness and diversity of sample type (Alpha diversity)

To compare the OTU diversity between sample approaches and sampling sites, we have calculated the alpha diversity index. There were two key points we were interested in. First, we wanted to know if the alpha diversity of the BAL samples was higher or lower than the diversity of the lung tissue samples. A larger or comparable alpha – diversity index would indicate that the BAL samples communities provide a representative snapshot picture of the microbial composition of the lung. However a lower alpha-diversity of the BAL samples would make functional assumption based on the BAL sampling difficult since a significant amount of taxa will not be described. Secondly, we expected that host cell removal from the BAL-minus material would reduce the diversity index because some bacteria could be stronger attached to the pulmonic cell surface than others and could be removed from the sample by centrifugation.

**Figure 2:**
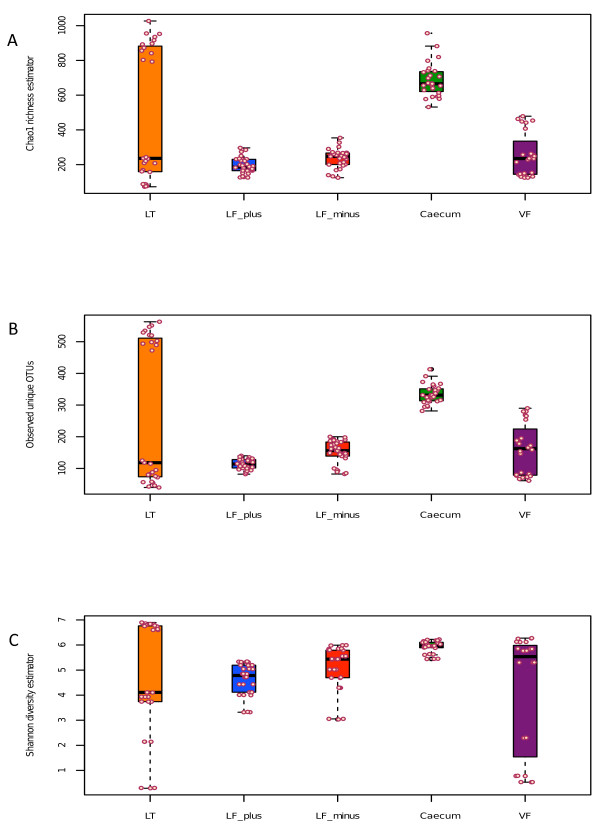
**Alpha diversity plots. A**: Chao1 richness estimator between sample types and individual samples (circles), LF-plus is bronchoalveolar lavage (BAL) fluids and LF-minus is BAL where the mouse cells have been removed. LT is lung tissue and VF is vaginal flushing, **B**: Observed unique OTUs and **C**: Shannon diversity estimator between sample types (s above) and individual samples (circles). The sequences (3350) were randomly even subsampled before calculating the alpha diversity. The boxplots show median, quartile, smallest and largest observations as well as outliers (circles). Significant variation is indicated by * (KW, p < 0.05).

The bacterial community of the BAL-minus were in 50% of the cases (indicated by the median) richer than the BAL-plus (Figure 2A). We found this difference to be significant (W, p < 0.05).

There was no significant variation between the BAL-minus and lung tissue samples. The mouse caecum community is generally richer than all other tested communities, except of the upper quartile of the tissue samples. The vaginal microbiota appeared to be as rich as the lung tissue community.

In more than half of the BAL-minus samples, more unique OTUs were observed than in the lung tissue material (Figure 2B). The BAL-plus samples contained significantly less OTUs than the BAL-minus samples (W, p < 0.001). The variation of Chao1 and observed OTUs comparing all pulmonic samples were significant (KW, p < 0.01)

We observed the highest number of unique OTUs in the caecum samples, compared to vaginal and lung tissue microbiota (W, p > 0.05).

A slightly different picture was observed for the diversity index (Figure 2C). In most cases the alpha diversity of BAL-minus samples appeared to be larger than the BAL-plus and lung tissue samples. However, the variation of diversity between all pulmonic samples was not significant (KW, p > 0.05). The Shannon index varied significantly when comparing both BAL-plus and BAL-minus communities only (W, p < 0.05) and reflect the observation of Chao1 and unique OTU sequences.

In summary, the mouse cell-free BAL samples yielded a richer microbial community, had a larger alpha-diversity and contained more unique OTU in comparison to the samples with mouse cells. In addition, at least 50% of the alpha-diversity observations the BAL-minus show larger diversity indexes than the lung tissue samples. The upper quartile of the lung tissue samples varied largely for all three diversity indicators approaching larger diversity (Shannon), richness (Chao1) and observed OTUs as found for the caecum samples. This could be the result of non-proper flushing or contamination during the experimental process. However, the low diversity, richness and fewer OTUs in the lung tissue samples correspond to higher diversity, richness and more OTUs in the matching BAL samples. There is also a large overlap in beta-diversity based on OTU abundance of lung tissue samples with the BAL samples, suggesting that, a biased flushing is more likely to be the reason, than contamination.

### Bacteria found via traditional culturing of BAL

To establish any possibly cultivable part of the lung microbiota and possible viable contaminations, we performed a conventional cultivation study of BAL fluids from 10 additional mice. Of the 40 different agar plates under various conditions with 200 μL BAL per plate from each of the 10 mice, we only found a few bacterial colonies on 5 plates originating from only 4 different mice. These bacteria colonies were all identified to be *Micrococcus luteus* with 99% probability by the Vitek2 system (Bio Mérieux, France).

## Discussion

### Methodology

In this work we have sequenced the lung bacterial 16S rRNA gene variable region V3/V4 with different methods and compared the results to gut and vaginal bacterial microbiome. We chose the V3/V4 region since Claesson et. Al [21] reported that it taxonomically characterizes microbial communities best without sequencing the entire 16S rRNA gene. Furthermore the same approach has been applied in multiple studies to study bacterial interaction with lakes, plants, humans and most important with mice [22-25].

In contrast to the general assumption, our results suggest that the lower airways in mice are not sterile and have a distinct bacterial microbiome that could probably influence airway diseases. A classic obstacle in the investigation of the microbiota of the lungs is the likelihood of contamination with bacteria from the upper respiratory tract (URT). This is especially true for the study of the human respiratory microbiome, because the procedure used has a high risk of contamination with oral microbiota [7]. In our study, this is bypassed by the invasive entry via the throat into trachea. We have extracted bacterial DNA from lung tissue, BAL with and without mouse cells and vaginal flushings. Our results show that it is possible to consistently obtain comparable sequences from the BAL fluid to use for community studies related the development of inflammatory disease in our mouse model.

The use of BAL as the sample for investigations has several advantages. The BAL sampling resembles the procedures used in humans, except that the work in animals bypasses both URT and oral microorganisms and samples the entire lung instead of just a local lung compartment. The microbial community has been shown to vary with the site of sampling in excised lung from a COPD lung transplant [26]. The removal of mouse cells in the BAL has the added advantage that we can use the lung cells and determine the lung inflammatory status of the mouse by staining and differentiating the immune cells [27]. A limitation of our study is the lack of comparison of our sequences with that of the upper respiratory flora. This could possibly be obtained by performing 16S rDNA sequencing on a matching nasal lavage sample for each mouse. This should be done in the future.

Our lung tissue samples showed some clustering that could indicate a sampling problem. In our study we sampled the distal tip of the left lung lobe after the BAL procedure was performed. The clustering could be a result of this BAL procedure not being equally effective between samples in the very low airways, sometimes leaving the distal tip un-flushed. This would predict a clustering showing one community equal to the one found in the BAL and one more rich and diverse representing the less rinsed tissue. If we were especially interested in the tissue associated microbiota, BAL should not be performed before sampling and mouse cells should not be removed from the BAL fluid before extraction.

Our results show that there are fewer OTUs in the BAL-plus samples with mouse cells and that the lung tissues samples have a large variation. This suggests that the removal of tissues and host cells is a viable approach, when extracting DNA for the examination of the lung microbiome.

Another challenge when working with low bacterial loads is the risk of contamination from the environment or sampling procedures. Some contamination must be expected and taken into account when interpreting data. We believe that we have taken large precautions to insure sterility during procedures and we have used excess controls to check that our sampling procedure or experimental chemicals did not produce any sequences on their own in the PCRs. Culturing of the BAL used for DNA extraction did not yield many bacteria either. Furthermore, our sequences were very consistent between mice. This would suggest that any contamination was either negligible or at least distributed evenly between mice.

We did find large variation within the vaginal samples resulting in subclustering into groups we designated S1 and S2 (Figure 1C and D). S1 (vaginal samples 2, 5 and 8) was found to be much more distantly related to caecum and lung communities than the S2 group, which more closely resembled the lung microbiota. We believe this could be the result of a possible infection in the S1 vaginas, as these 3 samples contained 56-97% *Streptococcus.* In the present study, we did not monitor the stage of the estrous cycle at the time of sampling, which has been shown to change the bacterial profile of the vagina in animals and humans [28,29]. Mice have a daily fluctuation in estrous cycle, which in part could explain the subclustering of the vaginal microbiota. This should be taken into account in futures studies.

There was a concern that the vaginal sampling procedure would yield a large overlap in OTUs with the gut microbiota due to cross contamination during sampling. This has been shown not to be the case, as we show here there is a very little overlap between caecum and vaginal microbiota. To our best knowledge this is the first time that the BALB/c mouse vaginal bacterial community has been investigated with 454 Pyrosequencing for a full community study. This promises to be useful in futures studies of the “inheritance” of bacterial microbiome from mother to pup or vaginal microbiome related diseases such as vaginosis [28,30].

We faced two main obstacles: The low DNA concentration in all samples, except for the caecal material and unspecific primer binding in the tissue samples.

To overcome the low DNA concentration we increased the PCR cycle number. The large cycle number essentially could amplify any kind of contamination or primer bias such as chimeras, but we adjusted the rounds of cycles to the crucial experimental negative controls as described in the material and methods. Our results are confirmed by the observed community profile of previous human lung observations (discussed in detail below) and the low abundance of chimera (<3% of quality trimmed sequences) [31,32].

The second obstacle was the non-specific binding of the primers in the lung tissue sample caused by the low amounts of bacterial DNA and large amounts of eukaryotic nucleic acids. Since the risk of contamination barely left space for adjustments, we chose to do a nested PCR and amplified a ∼ 1500 bp long fragment of the 16S rRNA gene prior amplifying the hyper variable region V3/V4. Although both primer sets are universal and theoretically target all bacteria and archaea, the tendency to favor certain taxonomic groups cannot be excluded, thus one primer set should be preferred to compare the different samples.

Therefor we were expecting a significantly different clustering in beta-diversity of the lung tissue community in comparison to the BAL fluids. However the differences were small supporting our methodology.

### The lung has a distinct bacterial community

It is not known from where we obtain our putative bacterial lung microbiota however it is most likely to be in a flux state with the environment. There is support for this notion in the hygiene hypothesis of the development of asthma and allergies [33]. We hypothesize that mice obtain the bacteria from their local environment and littermates influenced by handling by human, feed and water. But it is also a possibility that the core lung microbiome is established *in utero*, during and after birth in the very early life, as it is suggested with gut microbiota from human and animal studies [34-36].

The lung microbiota found via the culture independent methods described in our study of BALB/c mice, have been shown not to be just a subpopulation of the bacteria found in caecum samples and did not vary significantly between mice suggesting that a distinct lung microbiome exists. An example of this would be the sequence for *Pelomonas* 4818 (OTU ID), which was found in all our lung samples but not in any caecum samples. We did find 6 major genera that varied significantly between our different sampling methods for the lung bacterial community (KW, p < 0.05) (Additional file 5: Figure S3). *Acinetobacter*, *Pelomonas* were most abundant in the BAL-plus, where both *Acinetobacter* and *Pelomonas* have been associated with the human lung microbiota [4]. *Arcobacter* mostly found in BAL-minus has likewise been found to also be correlated with protection from skin allergy and protection from OVA allergy in mouse models [37-39] and found in human lungs [40]. *Polaromona, Schlegella* and *Brochothrix* have not previously been found in BAL fluids from humans or mice and are considered environmental bacteria. We have found *Prevotella* and *Veillonella* spp. only in the lung and vaginal samples. These species have been suggested to be a distinct part of lung microbiome and mucus epithelia in humans and the absence of *Bacteroides* associated with asthma [3,41].

We have also compared the genera variation of vaginal cluster S1 and S2 against all lung samples. S1 varied significantly in 4 taxa (Figure 1C and D) Genera observed <50 sequences sum counts were not considered. This cut off value was chosen as an additional denoising criterion necessary for sequences with high PCR cycle number. *Staphylococcus* was more abundant in the pulmonic samples (KW, p < 0.05) than in S1. Also, *Anaerococcus* and *Massilia* were not observed in the S1 samples. The large abundance of *Streptococcus* in S1 (KW, p < 0.05) varied clearly from the lung samples. The vaginal cluster S2 with high similarity in beta diversity towards the lung samples varied in 32 genera, but all taxa added up to less than our chosen detection minimum of 50 sequences.

### List of bacteria with possible influence on lung immunity

We wanted to identify the microbiota that possibly could influence lung immunity in our animal model. We created a list of interesting bacteria (prior to sequencing) at the genus, family or species level, based on other previous studies of both, human lung and animal models of disease. This list is found in Additional file 2: Table S2 and Additional file 6: Table S3. From our results we found bacteria associated with asthma and COPD in the mouse lung microbiome such as *Lachnospiraceae* and *Akkermansia muciniphilia*[42] and *Shewanella, Comamodacea*[43], *Haemophilus, Streptococcous, Fusobacteria*[3]. No indications were observed for *Bartonellaceae*, *Globicatella*, *Ralstoniacea* nor *Nitrosomonadaceae* from our premade list. No OTU sequence blasted could be assigned to *Clostridium difficile*, *Pseudomonas aeruginosa*, *Lactobacillus* OTU 1865, *Bacteriodales* OTU 991 or *Micrococcus luteus* from our list either. Sequences from the genera *Micrococcus* we isolated did not contain enough taxonomic information to differentiate between *Micrococcus luteus* found on our agar plates and other *Micrococcus* species.

The putative *Akkermansia muciniphilia* was found in lung and in one caecum sample and is especially interesting as it is a mucin degrading bacterium and has been shown to influence gut mucus layer thickness [44]. Recently, it was reported that *Akkermansia muciniphilia* is present in BALB/c caecum but not in fecal samples. The overall BALB/c caecal microbiome found in our study is also confirmed with the dominant phyla being *Firmicutes* (69.99%) and *Bacteroidetes* (22.07%) [45]. The presence of *Akkermansia muciniphilia* in the lung mucus layer could be of importance in asthma characterized by thickening of the epithelium and increased mucus production [46]. Most of the lung-associated bacteria that we identified in Additional file 2: Table S2 could only be found in the mouse lung and vagina samples but not in the caecum.

*Bifidobacterium animalis subsp. lactis*, and *Lactobacillus acidophilus NCFM* were added to the list of interesting species because of their use as probiotic bacteria in various mouse models and humans, and it would be interesting to know whether or not these bacteria are present in an unchallenged model. We found OTUs matching *Bifidobacterium animalis subsp. lactis, Bifidobacterium longum subsp. longum* and *Lactobacillus reutieri* the latter two not being on our list, in lung samples, but not in any caecum samples. *Bifidobacterium longum subsp. longum* have been found in human (meconium) and is regarded as one of the first colonisers of the gut originating from the mother [36]. Several strains of *Lactobacillus* have been shown to modulate allergic pulmonary inflammation, whereas *Lactobacillus reuteri* has been shown to reduce inflammation in BALB/c mice [47,48].

### Impact on animal models of inflammatory lung disease

The influence of gut microbiota on lung immunity has been vastly explored and several studies have linked changes in the gut microbiome with changes in lung immunity in mice [42,49-51]. As it is becoming clear that the microbiome of the animal used in a particular model influences that animal’s immune status and ultimately affects the outcome of experiments, it is important to take precautions in the model design. Things known to influence gut microbiome composition in laboratory mice include probiotics, antibiotics, stress, handling, vendor/site of breeding and animal lineages [52-55] and it is possible that these factors will affect the lung microbiota as well. Most studies done on gut microbiota and lung immunity do not take lung residing bacteria into account when the data are interpreted. It is possible that the local lung effects seen could be the results of changes in the lung as well as in the gut. In our studies we always use age matched female mice from the same site of breeding (lot number) and distribution of the mice equally between groups as to avoid any littermate bias. It should possibly also be noted whether or not the mice have mothers that are sisters or not as this also effects gut and possibly lung microbiome in the offspring [52]. As an added layer of complexity we should remember that the total mouse microbiota do not only consists of bacteria but also fungi and viruses. In particular bacteriophages could influence gut or lung microbiology and indirectly have adverse effects on health [56].

Future studies into the lung microbiota of mice should include a comparison between nasal lavages and BAL to distinguish between upper and lower respiratory tract microbiota and possibly longitudinal studies with culture independent techniques.

## Conclusions

BALB/cj mice were shown to have a lung microbiome that was distinct from their caecal but overlapping with their vaginal bacterial community. We have consistently amplified bacterial DNA from mouse BAL fluid and have shown that host DNA present in the DNA extraction step influences the community profiles obtained and that this needs to be taken into account when choosing methods, performing the analyses and prior to biological interpretation. Mouse models provide the means to obtain mechanistic insights into the lung microbiome. We believe that the lung microbiota should be considered when working with these mouse models of human disease and further research is needed to reveal the contribution of the lung microbiota to the pathogenesis of diseases such as respiratory disease common in infants (i.e. RSV), cystic fibrosis, COPD and asthma.

### Availability of supporting data

All supporting data are included as additional files and all sequences used in this study are available in the NCBI Sequence Read Archive under study accession number SRP033710 (http://www.ncbi.nlm.nih.gov/sra).

## Competing interests

The authors declare that they have no competing interests.

## Authors’ contributions

KKB conceived and designed the study, carried out the animal work and DNA extractions, and drafted the manuscript. MR did the 16S data generation, analysis and participated in the design of the study and manuscript. SSC performed the cultivation and bacterial identification. KAK, LHH, STL and SJS participated in design and coordination and helped to draft the manuscript. All authors read and approved the final manuscript.

## Supplementary Material

Additional file 1: Figure S5 Rarefaction curves. (A) Observed species – raw data. (B) Observed species after random even subsampling. The data shown in (A) accounted for all sequences generated. The graphs evened out after approx. 2000 sequences observed and revealed that the random even subsampled OTU table (B) at a sequencing depth of 3530 will be efficient to include also the rare OTUs. The subsampled OTU table (B) was used for the statistical analysis of this study and is the basis of the Figures 1 and 2. Click here for file

Additional file 2: Table S2 List of interesting taxa. This list shows the distribution of lung associated taxa between sampling methods and sites. Most of the lung-associated bacteria could only be found in the lung and vagina samples but not in the caecum. LF-plus is bronchoalveolar lavage (BAL) fluids and LF-minus is BAL where the mouse cells have been removed. LT is lung tissue,VF is vaginal flushing and caecum from the gut microbiota. Click here for file

Additional file 3: Table S4 Distribution of genera between samples. This table shows the distribution of genera between sample types. The observations are based on the summarized subsampled OTU table (3318 OTUs) after singletons and doubletons were removed. We discriminated between shared and unique genera of lung, vaginal and caecal environment. Click here for file

Additional file 4: Figure S4 Additional PCoA 2 and 3. The axis of PCoA plot 2 and 3 explain the 6.28%/24% and 10.42%/6.28% of the variances respectively. Both plots show the large overlap of bronchoalveolar lavage (BAL) fluids BAL-plus with mouse cells in BLUE, BAL-minus (without mouse cells) in RED and lung tissue in ORANGE and support plot 1. Only in plot 3 the caecal GREEN community overlaps with the lung and vaginal community confirming its large distance from the other sample sites. Click here for file

Additional file 5: Figure S3 Variation in lung genus composition. The genera shown counted up to at least 50 or more sequences in relative abundance and vary significantly among the lung communities (KW, p <0.05). LF-plus is bronchoalveolar lavage (BAL) fluids and LF-minus is BAL where the mouse cells have been removed. LT is lung tissue, VF is vaginal flushing and caecum represents gut microbiota. Click here for file

Additional file 6: Table S3 Blast search – putative species identity. For further identification the representative sequence of each OTU of the Qiime pipeline output were picked and blasted. OTUs were only considered when the highest score, maximum identity and 100% query cover uniquely matched one species. Additional subspecies information corresponds to the best hit. It is also noted from how many different animals and from which sampling site the OTUs were found. LF-plus is bronchoalveolar lavage (BAL) fluids and LF-minus is BAL where the mouse cells have been removed. LT is lung tissue, VF is vaginal flushing and caecum from the gut microbiota. Click here for file
