# Supplementary figures and images for: The murine lung microbiome in relation to the intestinal and vaginal bacterial communities
Source: BMC Microbiol. 2013 Dec 28;13:303. doi: 10.1186/1471-2180-13-303 (PMC3878784; doi:10.1186/1471-2180-13-303)

A

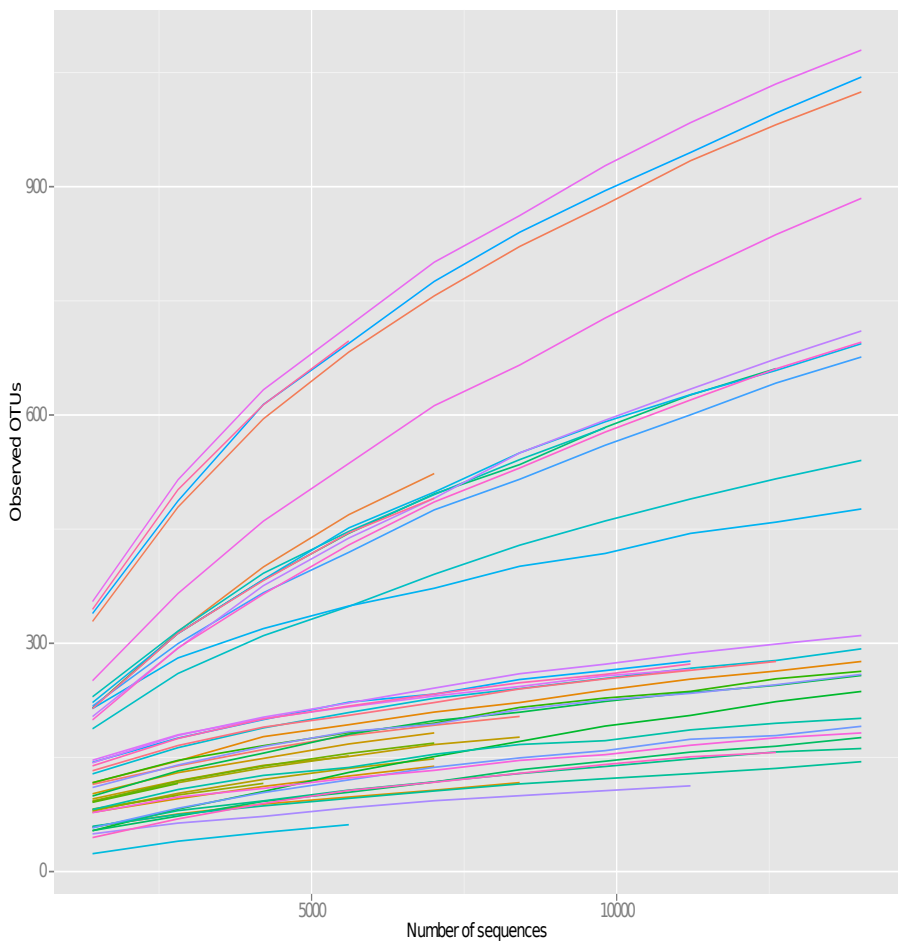

B

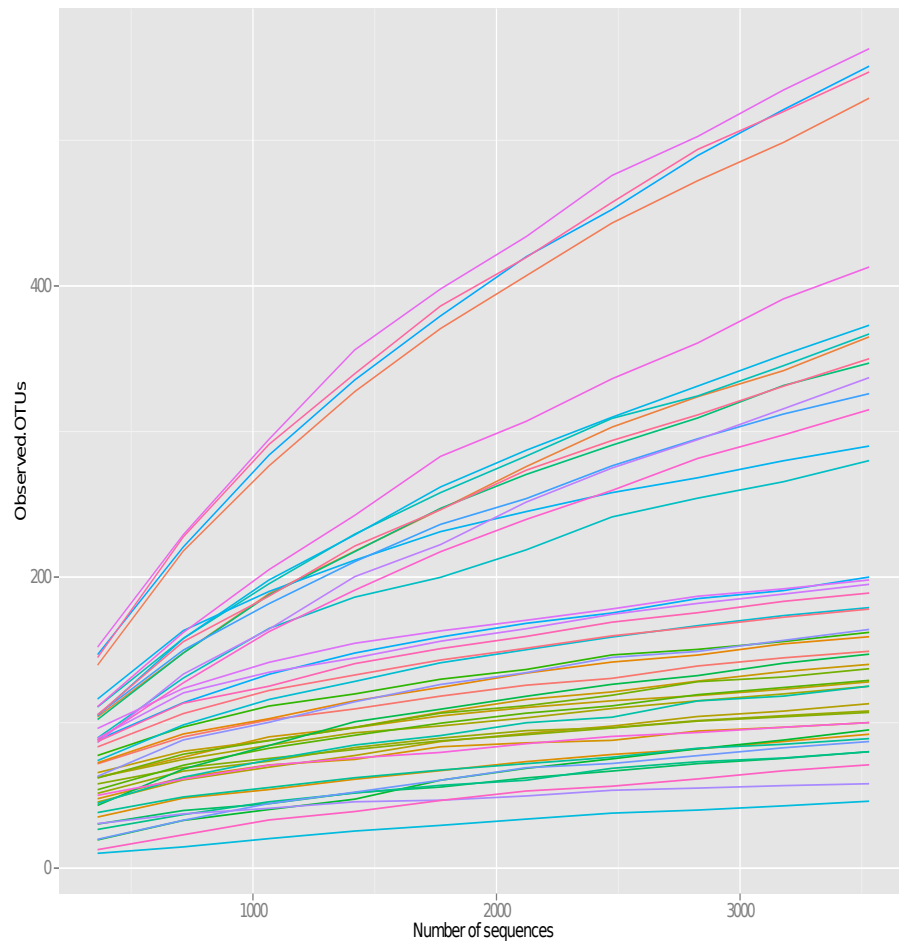

Supplement: Additional file 1: Figure S5 — Rarefaction curves. (A) Observed species – raw data. (B) Observed species after random even subsampling. The data shown in (A) accounted for all sequences generated. The graphs evened out after approx. 2000 sequences observed and revealed that the random even subsampled OTU table (B) at a sequencing depth of 3530 will be efficient to include also the rare OTUs. The subsampled OTU table (B) was used for the statistical analysis of this study and is the basis of the Figures 1 and 2. [file 1471-2180-13-303-S1.pdf]

*Acinetobacter*

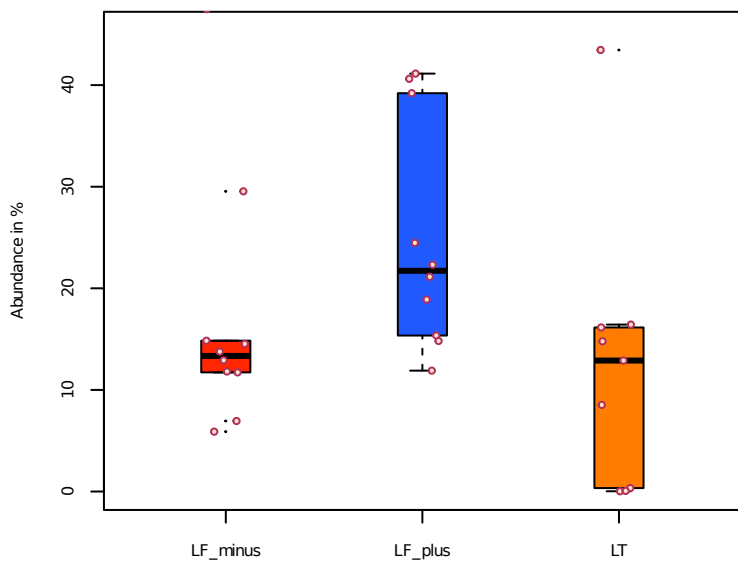

*Arcobacter*

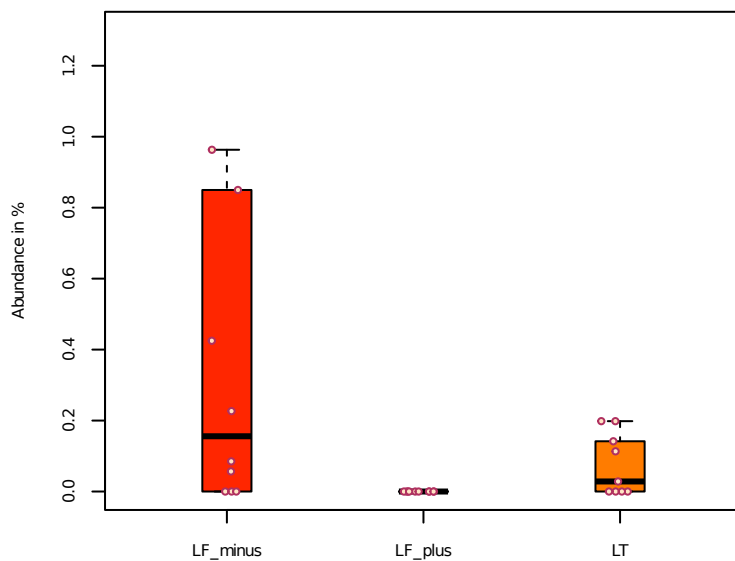

*Brochothrix*

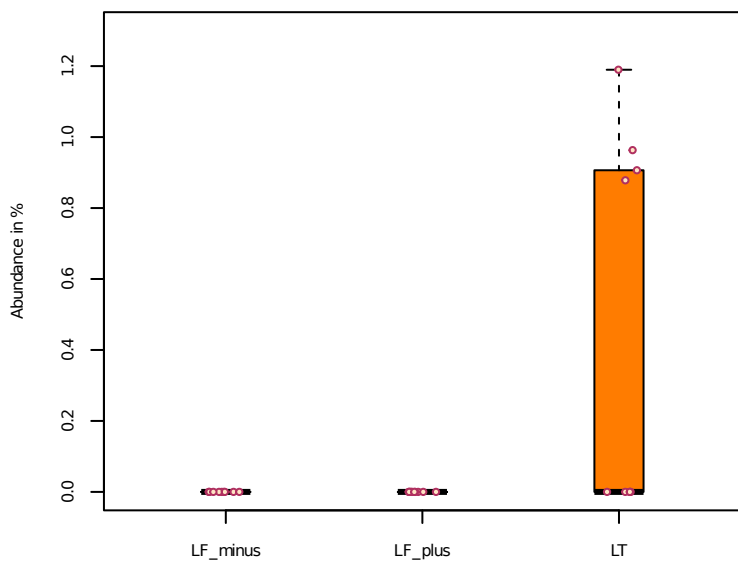

*Pelomonas*

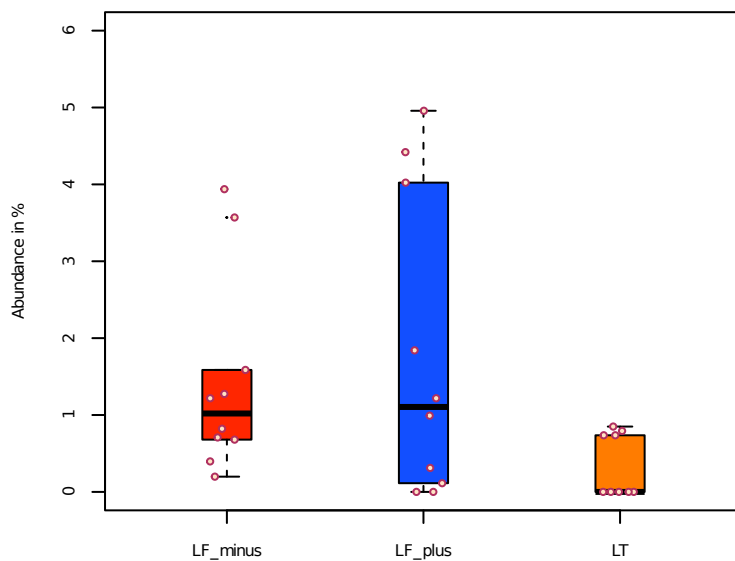

*Polaromonas*

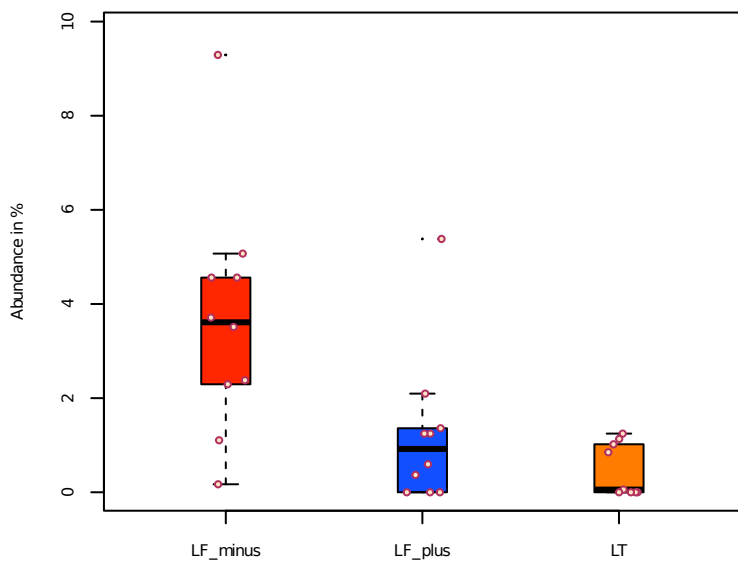

*Schlegelella*

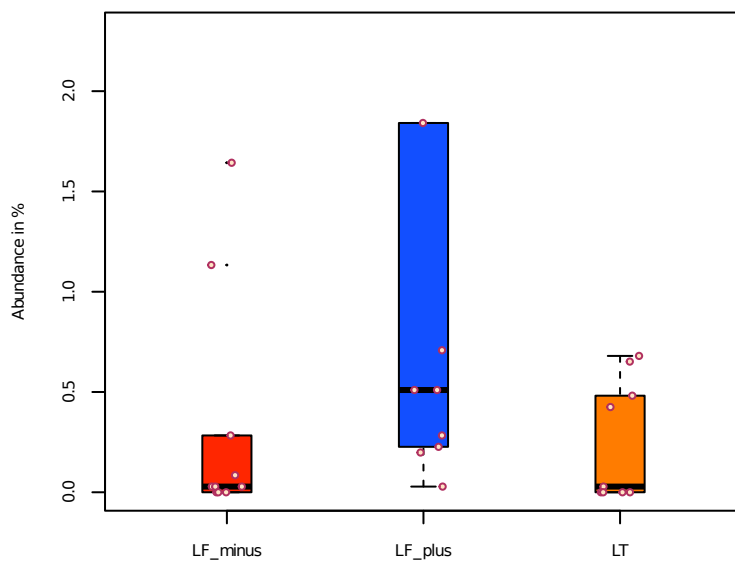

Supplement: Additional file 5: Figure S3 — Variation in lung genus composition. The genera shown counted up to at least 50 or more sequences in relative abundance and vary significantly among the lung communities (KW, p <0.05). LF-plus is bronchoalveolar lavage (BAL) fluids and LF-minus is BAL where the mouse cells have been removed. LT is lung tissue, VF is vaginal flushing and caecum represents gut microbiota. [file 1471-2180-13-303-S5.pdf]
