# Supplementary material for: The murine lung microbiome in relation to the intestinal and vaginal bacterial communities
Source: BMC Microbiol. 2013 Dec 28;13:303. doi: 10.1186/1471-2180-13-303 (PMC3878784; doi:10.1186/1471-2180-13-303)
Supplement: Additional file 4: Figure S4 — Additional PCoA 2 and 3. The axis of PCoA plot 2 and 3 explain the 6.28%/24% and 10.42%/6.28% of the variances respectively. Both plots show the large overlap of bronchoalveolar lavage (BAL) fluids BAL-plus with mouse cells in BLUE, BAL-minus (without mouse cells) in RED and lung tissue in ORANGE and support plot 1. Only in plot 3 the caecal GREEN community overlaps with the lung and vaginal community confirming its large distance from the other sample sites. [file 1471-2180-13-303-S4.pdf]

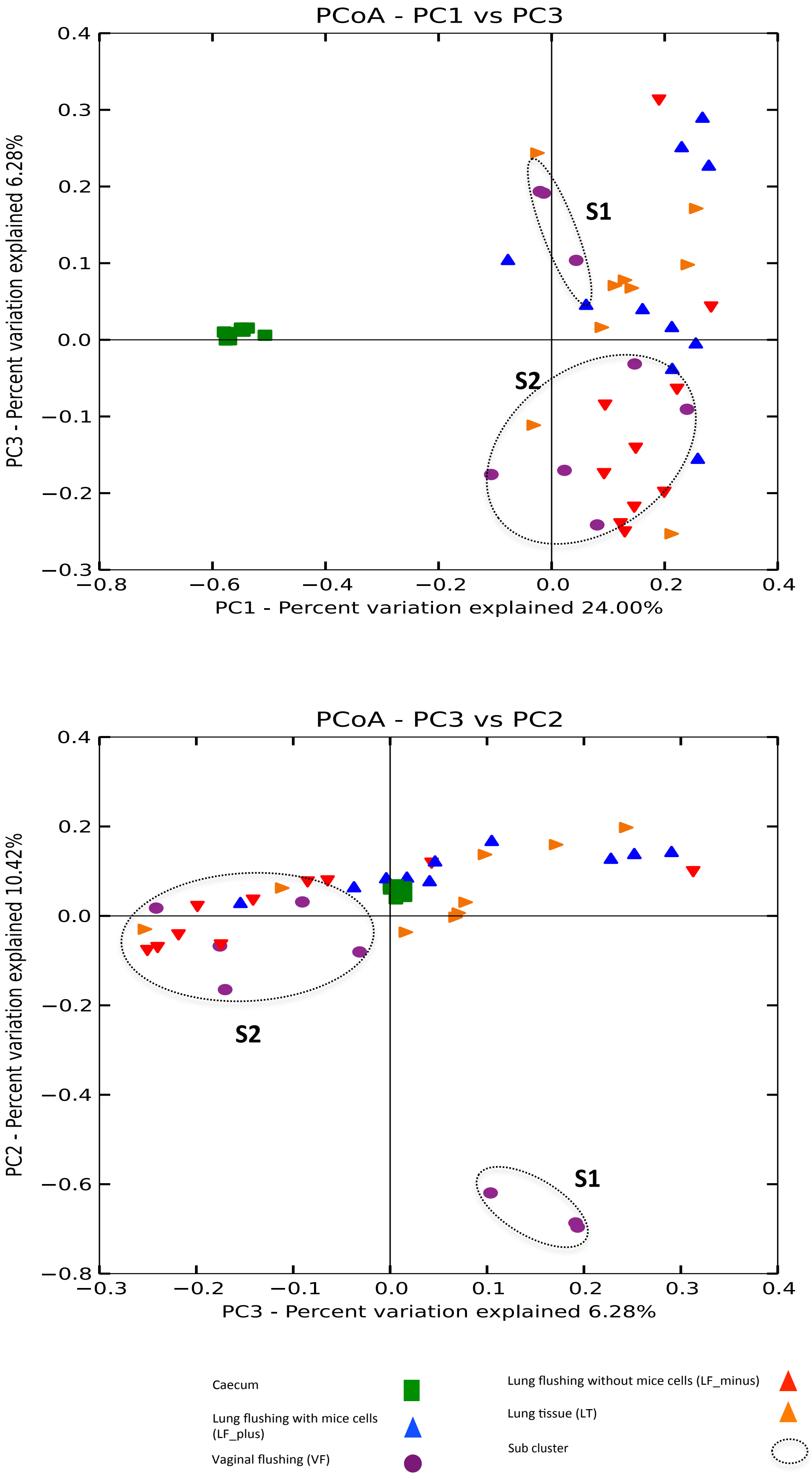
PCoA - PC1 vs PC3
PC3 - Percent variation explained 6.28%
PC1 - Percent variation explained 24.00%
S1
S2
0.4
0.3
0.2
0.1
0.0
−0.1
−0.2
−0.3
−0.8
−0.6
−0.4
−0.2
0.0
0.2
0.4
PCoA - PC3 vs PC2
PC2 - Percent variation explained 10.42%
PC3 - Percent variation explained 6.28%
S2
S1
0.4
0.2
0.0
−0.2
−0.4
−0.6
−0.8
−0.3
−0.2
−0.1
0.0
0.1
0.2
0.3
0.4
Caecum
Lung flushing with mice cells (LF_plus)
Vaginal flushing (VF)
Lung flushing without mice cells (LF_minus)
Lung tissue (LT)
Sub cluster
